# Histological evaluation of bone graft healing in maxillary sinus floor augmentation at two healing time points: a randomized clinical trial

**DOI:** 10.1590/0103-644020256560

**Published:** 2026-02-02

**Authors:** Ricardo Pasquini, Leandro Eduardo Klüppel, Rubens Moreno de Freitas, Luíz Antônio Violin, Silvio Roberto Consonni, Elcio Marcantonio, Tatiana Miranda Deliberador

**Affiliations:** 1 Department of Implant Dentistry at the Latin American Dental Research and Teaching Institute College(ILAPEO). CuritibaPR/ Brazil.; 2 Department of Biochemistry and Tissue Biology at the State University of Campinas- Institute of Biology (Unicamp-IB). Campinas Brazil; 3 Department of Periodontology, UNESP - University Estadual Paulista, Araraquara Dental School, Araraquara, São Paulo, Brazil

**Keywords:** Sinus lift, bone substitutes, bone transplantation, xenografts, dental implants

## Abstract

This study aims to compare the effects of two distinct healing periods (4 and 8 months) on bone formation after maxillary sinus floor elevation surgeries using sintered bovine bone grafts through histological and histomorphometric analyses. Using a split-mouth design, fourteen participants with bilateral edentulism in the posterior maxilla were included and randomized into two groups with 4 months (Test Group - TG) and 8 months (Control Group - CG) of healing periods. After the healing period, bone samples were collected and subjected to histomorphometric analysis to quantify the percentages of newly formed bone, residual bone substitute, and soft tissues. Comparison between the groups was performed using the paired Student’s t-test (p<0.05). Twenty-eight maxillary sinus grafts were performed, and 54 dental implants were placed. Histological analysis revealed newly formed trabecular bone tissue in close contact with the bone substitute surface, with higher thickness observed in the CG. Collagen fibers in the CG exhibited greater birefringence, indicating increased thickness and organization compared to the TG. Histomorphometric analysis demonstrated a significant difference in the percentage of new bone formation between the CG (28.22 ± 6.29%) and TG (20.02 ± 5.97%; p<0,001), as well as in the percentage of residual bone substitute (TG: 39.12 ± 8.15%; CG: 31.65 ± 8.70%; p = 0.005). The proportion of soft tissues remained stable between groups. The 8-month healing period significantly increased bone tissue formation and maturation, suggesting that this interval optimizes the quality of grafted bone when using sintered bovine bone substitute.

## Resumo

Este estudo tem como objetivo comparar os efeitos de dois períodos distintos de cicatrização (4 e 8 meses) na formação óssea após cirurgias de elevação do assoalho do seio maxilar usando enxertos ósseos bovinos sinterizados por meio de análises histológicas e histomorfométricas. Usando um delineamento de boca dividida, quatorze participantes com edentulismo bilateral na maxila posterior foram incluídos e randomizados em dois grupos com 4 meses (Grupo Teste - GT) e 8 meses (Grupo Controle - GC) de períodos de cicatrização. Após o período de cicatrização, amostras ósseas foram coletadas e submetidas à análise histomorfométrica para quantificar as porcentagens de osso neoformado, biomaterial residual e tecidos moles. A comparação entre os grupos foi realizada usando o teste t de Student pareado (p < 0,05). Vinte e oito enxertos de seio maxilar foram realizados e 54 implantes dentários foram instalados. A análise histológica revelou tecido ósseo trabecular neoformado em contato próximo com a superfície do biomaterial, com maior espessura observada no GC. As fibras de colágeno no GC apresentaram maior birrefringência, indicando aumento de espessura e organização em comparação ao GT. A análise histomorfométrica demonstrou diferença significativa na porcentagem de formação óssea nova entre o GC (28,22 ± 6,29%) e o GT (20,02 ± 5,97%; p < 0,001), bem como na porcentagem de biomaterial residual (GT: 39,12 ± 8,15%; GC: 31,65 ± 8,70%; p = 0,005). A proporção de tecidos moles permaneceu estável entre os grupos. O período de cicatrização de 8 meses aumentou significativamente a formação e a maturação do tecido ósseo, sugerindo que esse intervalo otimiza a qualidade do osso enxertado ao utilizar substituto ósseo bovino sinterizado.

**Figure:**
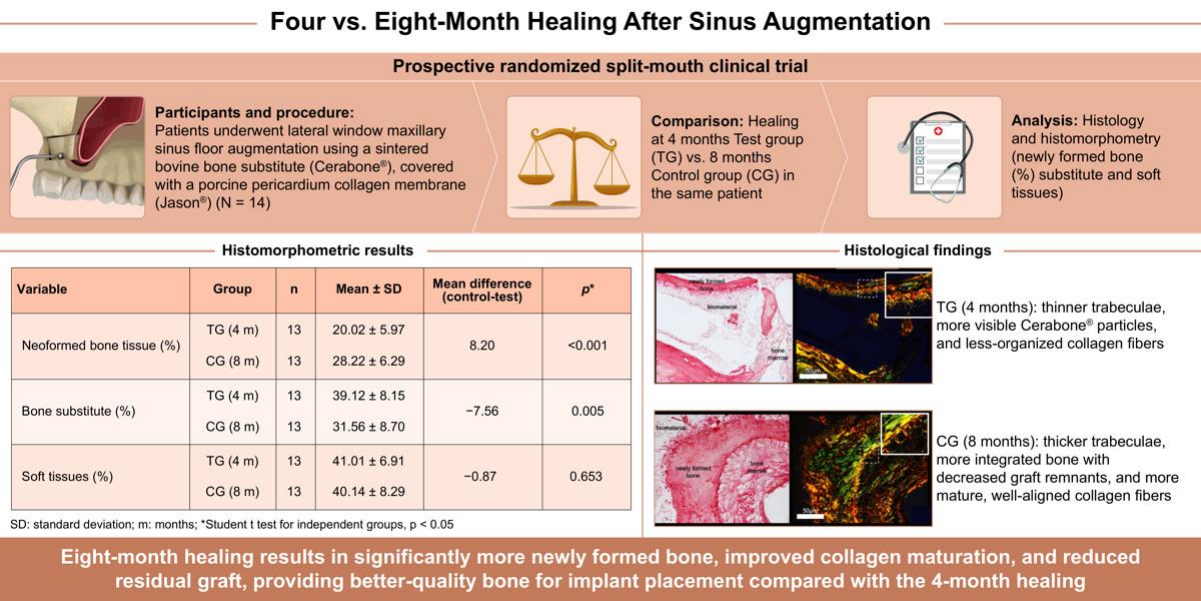

## Introduction

Tooth loss in the posterior maxillary region leads to physiological changes, such as bone resorption and maxillary sinus pneumatization, that preclude the placement of osseointegrated implants^1^. To overcome this limitation, various surgical strategies have been proposed, including maxillary sinus lifts, the use of short implants, tilted implants, and zygomatic implants ^2^ ^,^ ^3^ ^,^ ^4^. All these approaches have high success rates in implant-supported rehabilitations^5^.

Among these alternatives, maxillary sinus lifts are widely used to increase bone height in the posterior maxillary region, enabling the placement of conventional dental implants ^2^ ^,^ ^4^ ^,^ ^6^. Two surgical techniques are described for this intervention: the lateral window technique, introduced by Tatum (1977) and later published by Boyne (1980)^7^ ^,^ ^8^, and the transalveolar technique, proposed by Summers in 1996^9^. The lateral window technique is recommended when the residual alveolar ridge height is less than 6mm^10^. This procedure involves performing an osteotomy in the lateral wall of the maxillary sinus, allowing the displacement of the Schneiderian membrane and subsequent insertion of a bone graft in the created space. The graft plays a fundamental role in stabilizing the clot and maintaining bone volume, thereby favoring bone neoformation during osseointegration^11^.

Various bone substitutes, including autogenous bone, allografts, synthetic materials, and bovine bone substitutes, can be used for this purpose. They are widely used due to their osteoconductive capability and biocompatibility, which support tissue regeneration and maintain the elevated sinus membrane^12^. Depending on the type of graft used, after healing periods of 4 or 8 months, sufficient bone height can be obtained for regular implant placement, enabling subsequent implant-supported rehabilitation^13^.

Systematic reviews suggest that the survival rates of implants placed in areas grafted with xenogeneic bone are similar to those grafted with particulate autogenous bone^14^. Using xenogeneic grafts in maxillary sinus lift procedures is widely documented in clinical, histological, and histomorphometric studies^15^ ^,^ ^16^ ^,^ ^17^. However, there is a lack of studies regarding the optimal healing time for implant placement in areas grafted exclusively with sintered bovine bone substitutes. The duration of the healing period can significantly influence the quantity and quality of the neoformed bone, directly impacting the predictability of osseointegrated implants^18^.

Considering this, this split-mouth randomized clinical trial aimed to compare bone neoformation after maxillary sinus lift using sintered xenogeneic bone through histological and histomorphometric analyses in two distinct healing times (4 and 8 months).

The study hypothesis is that a more extended healing period (8 months) after maxillary sinus lift with sintered xenogeneic bone graft will result in better quality, quantity, and volume of neoformed bone, providing a more favorable environment for dental implant placement compared to a shorter healing period (4 months).

## Materials and methods

This prospective split-mouth randomized clinical trial was conducted in accordance with the Consolidated Standards of Reporting Trials (CONSORT) guidelines. The protocol was approved by the Research Ethics Committee of ILAPEO College under number # 4.894.128 (August 2021), CAAE 4687542.4.0000.9427.

The clinical stages were performed at the Implant Dentistry Clinic of ILAPEO College in Curitiba, Paraná, between September 2021 and July 2022. All participating patients were thoroughly informed about the study's objectives and procedures. They received appropriate verbal and written clarifications, and upon understanding the study's content and agreeing with the established terms, they signed the Informed Consent Form.

### Patient Selection and Sample Size

Fourteen patients of both sexes were selected, according to the following inclusion criteria: bilateral edentulism in the posterior maxillary region, with alveolar ridge height less than 6 mm, age between 30 and 80 years, and surgical indication for maxillary sinus lift without obstructions in the ostiomeatal complex.

Exclusion criteria included compromised systemic conditions, smokers or former smokers for less than 10 years, continuous use of alcohol or drugs, pregnancy or lactation, history of head and neck radiotherapy, use of bisphosphonates or immunosuppressants, platelet disorders, uncontrolled diabetes, chronic sinusitis, presence of sinus pathologies, and/or residual teeth in the maxillary sinus.

The sample size was calculated using the Power and Sample Size module of the Stata/SE v.14.1 software (StataCorp LP, USA). The calculation was based on data reported by Zhang et al. (2019)^19^, aiming to detect significant differences between the two treatments (4 and 8 months of healing) regarding the percentage of neoformed bone, residual bone substitute, and soft tissues. Considering an average difference between the two treatments of 5.40% with a standard deviation of 5.48%, a significance level of 0.05, and a test power of 90%, 13 cases were required to detect a significant difference between the groups.

### Randomization

The maxillary sinuses of the patients were randomly allocated into two groups: the Test Group (TG) with a healing time of 4 months and the Control Group (CG) with a healing time of 8 months, using a split-mouth design. Randomization was performed using a computer program (Microsoft Excel RAND). Each patient was considered an experimental unit, representing both their test and control groups.

### Surgical procedure for maxillary sinus graft

Before surgery, cone-beam computed tomography (CBCT) scans were performed to evaluate the anatomy of the maxillary sinus, residual bone volume, and obstructions of the ostiomeatal complex. All surgeries were performed by a single experienced surgeon (RPF) with two distinct healing times: 4 and 8 months. Maxillary sinus grafts were performed at the beginning of the study (month 0) for the Control Group (CG) and after 4 months for the Test Group (TG).

Patients were medicated with antibiotics (Amoxicillin 875 mg + Potassium Clavulanate 125 mg) twice a day for 7 days, starting 24 hours before the procedure. Moments before surgery, intra-oral antisepsis was performed using a 0.12% chlorhexidine solution as a mouthwash for 1 minute. Extra-oral antisepsis was performed with sterile gauze soaked in 2% chlorhexidine soap applied to the skin of the lower two-thirds of the face.

A local infiltrative anesthesia (4% Articaine with epinephrine 1:100,000; DFL Rio de Janeiro) was performed. A disposable 15c scalpel blade (Swann Morton®, United Kingdom) mounted on a straight scalpel handle was used to make an incision over the keratinized mucosa covering the alveolar ridge, followed by two diverging vertical relaxing incisions at the mesial and distal margins of the flap, allowing the elevation of a mucoperiosteal flap with a #24G elevator (Hu-Friedy®, Pennsylvania, USA) to expose the lateral wall of the maxillary sinus. The osteotomy was performed using a No. 6 diamond spherical bur mounted on a 2:1 angulated handpiece (Kavo do Brasil, Joinville -SC, Brazil) to create access to the maxillary sinus through a lateral window, following the technique described by Boyne (1980)^8^. The osteotomy began 3 mm above the sinus floor and 2 mm posterior to the anterior wall. One or two windows were created depending on the anatomy of the maxillary sinus in cases of present septa or voluminous sinuses.

After the osteotomy, the sinus membrane was observed, retaining the remaining cortical bone in the center. The Schneiderian membrane was carefully detached and elevated into the sinus cavity using specific sinus lift curettes (Kit Curetas SLA, IM3, São Paulo, Brazil). The space resulting from the membrane elevation was filled with sintered xenogenic bone substitute (Cerabone® - Straumann, Switzerland), containing granules of 1-2mm in size, hydrated with saline solution, carefully inserted into the posterior, medial, and anterior regions of the maxillary sinus, filling the cavity and avoiding excessive compaction.

Simultaneously, a resorbable native collagen membrane obtained from the porcine pericardium (Jason - Straumann, Switzerland) was positioned on the outer part of the lateral sinus wall and fixed with pins (Kit Strong TAC, IM3, São Paulo, Brazil). After the procedure, the flap was repositioned and sutured without tension using monofilament suture (Mononylon® 5-0, Ethicon®, New Jersey, USA).

Immediately after the procedure, postoperative medication was initiated with an anti-inflammatory (Ibuprofen 600 mg, three times a day for three days) and an analgesic (Paracetamol 750 mg, four times a day for two days). Postoperative instructions were provided to all patients, and sutures were removed between 10 and 14 days after the surgical procedure. The surgical area remained free from the direct load of the provisional prostheses on the tissues throughout the bone healing period. Patients were submitted to weekly postoperative follow-up clinical exams during the first month and until the eighth month before biopsy collection and implant placement.

### Biopsy collection

After 8 months of the maxillary sinus lift healing, a new CBCT scan was performed to identify appropriate sites for bone sample collection and implant placement planning. The procedure strictly followed the same internal and external asepsis protocols, anesthesia, incision on the ridge top, and mucosal elevation, all of which were performed in the initial phase of the sinus graft surgical protocol. Bone samples were collected using a trephine drill (Neodent®, Curitiba, Brazil) with an internal diameter of 3.5 mm and an external diameter of 4.2 mm, and a length of 17 mm, attached to a 20:1 reduction contra-angle handpiece (Kavo do Brasil, Joinville -SC, Brazil). One to two cylindrical bone samples were obtained from the grafted areas precisely at the planned implant sites. The highest-quality sample was selected from each patient, ensuring that each subject provided a single biopsy for analysis, regardless of the number of cylinders collected. The samples were collected in full thickness, covering the alveolar ridge crest to the height of the sinus membrane floor, including both residual alveolar bone and grafted bone.

To rehabilitate the study participants, Grand Morse Helix® implants (Neodent®, Curitiba-PR, Brazil) with 5.0 mm diameter and lengths ranging from 8.5 to 13 mm were placed at this surgical stage.

The insertion torque was measured using a manual torque wrench (Neodent®, Curitiba-PR, Brazil) during implant placement. The cover screws were screwed into the implants, and the flap was repositioned and sutured, keeping the implants submerged for healing. Patients were adequately instructed on pre- and postoperative medication and care. Six months after implant placement, patients underwent reopening to expose the implants to the oral environment, select and install prosthetic components, and undergo oral rehabilitation with implant-supported prostheses. The implant survival rate was evaluated one year after oral rehabilitation. Implant survival was defined as the absence of implant loss at each follow-up.

### Histological and histomorphometric analysis

Of the 28 trephine cores collected (inner diameter 3.75 mm; outer 4.30 mm), two were excluded due to technical artifacts, yielding 26 specimens (13 per group). Immediately after retrieval, the crestal/native-bone end of each core was marked with India ink to preserve orientation. Specimens were fixed in 10% neutral-buffered formalin (pH 7.4) for ≥48 h, decalcified at room temperature in an EDTA-based solution with regular changes (approximately 40 days), dehydrated in graded ethanol, cleared in xylene, embedded in paraffin (Paraplast®, Sigma-Aldrich, USA), and sectioned at 5 µm on a rotary microtome (Leica, Germany) in planes parallel to the residual bone-graft axis.

Each slide contained 4 to 9 serial 5-µm sections, depending on core length. For quantitative morphometry, one artifact-free section with sufficient grafted area was analyzed under blinded conditions (group allocation concealed). Sections were stained with hematoxylin-eosin (H&E) for quantitative analysis and with Sirius Red-F3B plus hematoxylin for qualitative collagen assessment (Montes, 1996). Slides were digitized on an Aperio CS (Leica) scanner; overview at ×10 verified orientation/integrity, and quantification was performed at ×20. Sirius Red sections were examined under polarized light at ×20 and ×40 using a Nikon Eclipse E800® microscope coupled to a P6FL® camera (Optika, Italy).

Within the grafted compartment of the analyzed section, one non-overlapping region of interest (ROI) of approximately 5.5 mm² was randomly selected by the morphologist, explicitly avoiding processing artifacts and immediate interfaces with native crestal bone and the sinus membrane. When anatomical constraints prevented an exact 5.5 mm² square, the exact ROI area was recorded for consistency and normalization.

Image analysis was performed in ImageJ (NIH) with calibrated pixel size (µm/pixel). Three tissue compartments were delineated using threshold-based masks with manual refinement by an experienced examiner (blinded): newly formed bone (NB), residual bone substitute (RB), and soft tissue/bone marrow (ST).

### Morphometric outcomes

Primary morphometric outcomes were expressed as area fractions (%) of NB, RB, and ST within a single, randomly selected ROI (~5.5 mm²) located in the grafted compartment and free of artifacts and immediate interfaces. The ROI area was recorded for each specimen to ensure consistency. By the Delesse principle, area fractions measured on 2D sections are unbiased estimators of volume fractions; therefore, percentages directly reflect tissue composition.

### Statistical Analysis

Each patient was considered an experimental unit, with one side representing the TG and the other the CG. The results were presented using descriptive statistics, including mean, standard deviation, median, maximum, and minimum values. The histomorphometric analyses included quantifying the percentages of neoformed bone tissue, remaining bone substitute, and soft tissues. Differences between the healing periods of 4 and 8 months were evaluated using the paired Student's t-test, with statistical significance (*p<0.05*). To compare the insertion torque between groups, the Student's t-test was applied.

Data were organized in an Excel spreadsheet and analyzed using IBM SPSS Statistics v.29.0 software. Quantitative variables were described by mean, standard deviation, median, and minimum and maximum values. Categorical variables were expressed in absolute and percentage frequencies.

## Results

Fourteen patients with bilateral edentulism in the posterior maxillary region, 10 women and 4 men, with a mean age of 52.9±16.1 (varying from 32 to 77 years), were included in this clinical study. All patients underwent bilateral maxillary sinus lifts.

Two patients from the CG had surgical complications, both presenting a small perforation of the Schneiderian membrane in one of the maxillary sinuses. The treatment involved applying a collagen membrane over the perforated area to achieve obliteration, allowing regeneration without compromising the graft and implant placement.

After 1 year of functional load, the implant survival rate was 98.1%, with only one implant lost in the CG before prosthesis installation. Patients with total edentulism were rehabilitated with hybrid protocol prostheses, while those with partial edentulism received fixed metal-ceramic prostheses.

### Histological analysis

Among the 14 patients initially included in the study (28 maxillary sinuses), one patient was excluded from the histomorphometric analysis due to inadequate sample quality. Thus, analyses were conducted on 13 patients, totaling 26 bone samples (13 from the CG and 13 from the TG).

The qualitative histological evaluation revealed the presence of neoformed trabecular bone tissue located within the bone substitute's interconnected porous areas and adjacent to bone substitute areas associated with soft tissues and remaining graft material particles. These characteristics were observed in samples analyzed after 4 and 8 months of healing.

**Figure 1:**
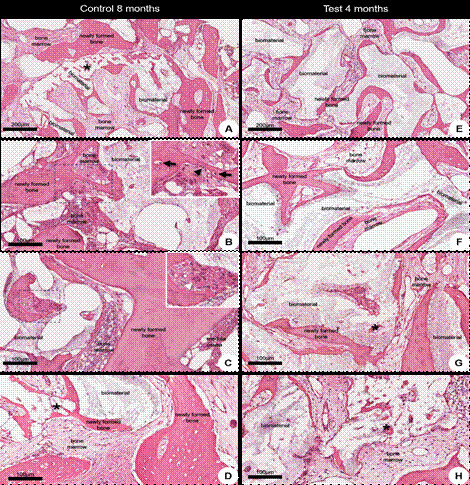
Representative photomicrographs of longitudinal sections of biopsies after 4 (A-D) and 8 (E-F) months of grafting. (A and E) At lower magnification, the trabeculae areas of neoformed bone tissue are around bone substitute areas or filled with bone marrow. It is possible to observe a difference in the thickness of these trabecular bones between the biopsies taken after 4 and 8 months of grafting. (B-D; F-H) At higher magnification, there is a flocculated bone substitute with an osteocyte nucleus (black arrow) within thin trabeculae of neoformed bone tissue. In a cross-sectional plane, these trabeculae permeate the interior of the region where the bone substitute is located (asterisk). In the bone marrow interface with the bone trabecula, there are bone lining cells, osteoblasts (black arrowhead), and osteoclasts (white arrowhead). Qualitatively, in the biopsies taken 8 months after grafting, the trabeculae of neoformed bone tissue are thicker than those taken 4 months after grafting. Furthermore, in both study groups, no gaps were found in the graft area without an osteocyte nucleus. Hematoxylin and Eosin (H&E) staining. Scale bars A and E = 200 µm; B-D; F-H = 100 µm.

Biopsies from the control group (CG) exhibited larger areas of bone tissue compared to the test group (TG) (Figure 1). The neoformed trabecular bone areas were narrow and varied in size, showing bone lining cells, osteoblasts, and osteocytes. Additionally, regions containing osteoclasts were observed at the interface between bone substitute and soft tissue in both groups (Figure 1, B-D; F-H). The neoformed bone tissue was in direct contact with the bone substitute, while osteoclasts were occasionally identified near the bone trabeculae in both groups (Figure 1G). The medullary spaces were filled with connective tissue, ranging from loose to dense unmodeled tissue, with no evident signs of active inflammatory processes in any of the analyzed samples.

**Figure 2:**
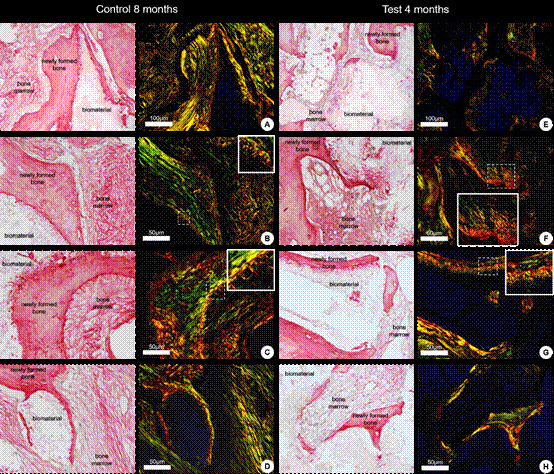
Representative photomicrographs of longitudinal sections of biopsies after 4 (A-D) and 8 (E-F) months of grafting under conventional light (left panel) and polarized light (right panel). (A and E) At lower magnification, the areas of trabeculae of neoformed bone tissue surround bone substitute areas or are filled with bone marrow. (B-D; F-H) At higher magnification, the organization of collagen fibers in the neoformed bone tissue in biopsies taken after 4 and 8 months of grafting. A progressive increase in collagen fiber thickness is observed as it transitions from orange and red to yellow and green, which is associated with mature collagen. The dark areas indicate a lack of collagen organization. Sirius Red and Hematoxylin staining. Scale bar A and E = 100 µm; B-D, F-H = 50 µm.

Analysis under polarized light revealed that collagen fibers presented birefringence, an optical property that modulates the direction of polarized light. This characteristic enabled the evaluation of the quantity, density, and degree of organization of collagen fibers. Birefringent areas were observed in the neoformed trabeculae bone in both groups (Figure 2). 

Qualitatively, the CG exhibited more intense birefringence in the green color, indicating a more advanced stage of the healing process, characterized by greater density, organization, and orientation of collagen fibers. Notably, collagen structures projecting perpendicularly to the neoformed bone trabeculae, extending towards the medullary tissue, were identified (Figure 2).

### Histomorphometric Analysis

**Figure 3:**
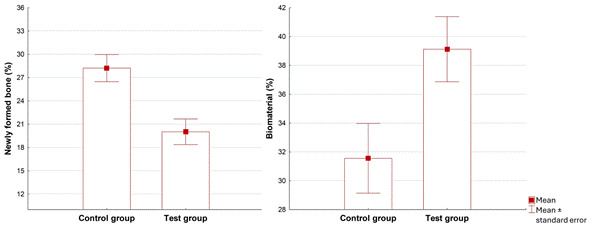
Histomorphometric analysis of the percentage of neoformed bone tissue and residual bone substitute between the groups.

The results of the histomorphometric analysis are presented in Table 1 and Figure 3. Statistical analysis revealed significant differences between the groups’ percentages of newly formed bone tissue and residual bone substitute. However, no significant differences were observed in the proportion of soft tissues. The Control Group (8 months) showed an increase in the amount of newly formed bone tissue and a reduction in the percentage of residual bone substitute compared to the Test Group (4 months). The percentage of soft tissues remained statistically similar between the groups.

**Table 1:** Histomorphometric results after 4 and 8 months of healing for the CG and TG.

Variable	Group	n	Mean ± sd	Median (min - max)	Mean Diff (control-test)	p*
Neoformed bone tissue	TG (4m)	13	20.02 ± 5.97	19.79 (11.66 - 36.00)	8.20	<0.001
(%)	CG (8m)	13	28.22 ± 6.29	25.25 (20.35 - 41.12)		
Bone substitute (%)	TG (4m)	13	39.12 ± 8.15	41.40 (26.63 - 54.27)	-7.56	0.005
	CG (8m)	13	31.56 ± 8.70	33.87 (6.47 - 40.21)		
Soft tissues (%)	TG (4m)	13	41.01 ± 6.91	40.89 (31.05 - 51.36)	-0.87	0.653
	CG (8m)	13	40.14 ± 8.29	40.22 (22.86 - 59.57)		

SD: standard deviation; m: months; %: percentage; CG: Control Group; TG: Test Group, Min: minimum; Max: maximum, Diff: difference

* Student t-test for independent groups, p<0.05

Figure 3 illustrates the results of histomorphometric measurements, showing the percentages of neoformed bone tissue and residual bone substitute in each group. In the CG, there was a significant difference in the rate of newly formed bone tissue (*p<0.05*), with a reduction in the proportion of residual bone substitute in the CG compared to the TG (*p=0.005*).

### Insertion torque

The insertion torque was measured in all implants placed in both groups. The mean insertion torque in the CG group was 38,4 ± 11,19 N.cm varying from 20 N · cm to 60 N · cm, while the mean insertion torque in the TG group was 35,1 ± 13,9 N.cm varying from 20 N · cm to 60 N · cm. No statistical difference was observed between groups (*p>0.07*).

## Discussion

The results of this study demonstrated that the bone substitute used was effective in maintaining the elevated sinus membrane, preserving the physical dimensions of the graft, and promoting its complete integration into a matrix of newly formed bone, thereby supporting the findings of Xu et al. (2004) ^22^. Additionally, the histological and histomorphometric differences observed suggest a more advanced degree of bone maturation around the particles of the sintered bovine bone substitute after 8 months of healing (CG), reinforcing the initial hypothesis of the study.

Previous studies evaluated the bovine xenogeneic bone substitute after a 6-month healing period. They found percentages of newly formed bone of 23.6%, 24.63%, 25.13%, and 35.4%, which are similar to those observed in this study after 8 months of healing ^18^ ^,^ ^23^ ^,^ ^24^ ^,^ ^25^. Tawil et al.^26^ specifically evaluated the effectiveness of Cerabone on sinus floor elevation, the same material and clinical condition analyzed in this study, after two different healing times. They found a newly formed bone of 22.77% ± 5.89% after 5.73 ± 0.44 months of healing and 26.15% ± 11.18% after 8.68±1.76 months of healing. It can be observed that the newly formed bone enhances over time, and the percentage of newly formed bone is similar to that observed in this study in both healing periods, 4 and 8 months.

A substantial claim of bone substitute is its ability to be resorbed and replaced by new bone at a speed that allows the bone regeneration in a clinically adequate time. It is well known that xenogeneic materials are resorbed very slowly and can remain in the graft area for years after the procedure. Despite its slow resorption, this material is recognized as a good option for sinus grafting^26^. The Cerabone used in this study remained in the graft area after 8 months of healing at a rate of 31.56%, which is similar to that found in the literature ^18^ ^,^ ^26^. Additionally, a significant difference in the percentage of residual bone substitute was observed, likely associated with graft compaction, graft consolidation, contraction, and maxillary sinus re-pneumatization^27^.

The Sirius Red staining, analyzed under polarized light microscopy, revealed birefringent areas in the neoformed trabecular bone of both groups. In the CG, the birefringence was more intense, with green shades indicating an organized collagen fiber pattern characteristic of concentric lamellar bone, which is typically associated with mature bone. In the TG, there was a gradual increase in collagen fiber thickness, with transitions from orange/red to yellow shades, indicating less intense birefringence and a disorganized fibrillar arrangement of collagen fibers distributed in different directions, characteristic of immature bone formation.

These findings align with Danish-Sani et al.'s ^6^ systematic review 6, which evaluated the effect of healing time on bone maturation of different bone substitutes in maxillary sinus grafts based on histomorphometric analyses. The review indicated that healing times longer than 6.22 months resulted in more bone formation than shorter periods (≤ 4.5 months). The present study corroborates these findings, demonstrating that after 8 months of healing, collagen fibers have greater density, organization, and orientation, suggesting a more advanced stage of bone maturation. In contrast, at 4 months of healing, the formed bone exhibits characteristics of immaturity, indicating that the collagen fiber synthesis process is still in its initial phase.

In this study, despite the significant difference between the analyzed groups, this healing pattern did not compromise the early implant placement, as the insertion torques were similar between the groups (TG: 35.1 N · cm +/-13.9; CG: 38.4 N · cm +/- 11.19). Furthermore, the high implant survival rates in both groups (98.1%) indicate that maxillary sinus grafting with sintered xenogeneic bone substitute is viable for implant placement at 4 and 8 months of healing. Although histological differences were identified in the bone maturation process between the two periods, the implant insertion torques did not show statistically significant differences. Additionally, all implants underwent a 6-month osseointegration period, followed by a prosthetic rehabilitation phase for each case.

Several critical factors influence the decision to anticipate implant placement after only 4 months of healing. The quantity and quality of residual bone, along with the implants’ geometry, design, and surface treatment, are fundamental determinants of achieving adequate insertion torque and effective initial stability, ensuring higher success and survival rates of the implants ^28^. The choice of early implant placement during the graft maturation phase, typically occurring around the fourth month, can provide significant benefits to the patient. This approach not only stimulates the grafted area but also allows the osseointegration process of the implant to coincide with graft maturation, which typically reaches a viable condition in the eighth month of healing. As a result, this strategy can reduce the interval between the surgical and prosthetic phases by 4 months, promoting clinical gain for the patient.

One limitation of this study is the time to analyze bone substitute resorption. As xenogeneic materials are resorbed more slowly, it is essential to monitor their resorption over a more extended period to understand the outcome.

Future implications of this study include the possibility of revising healing protocols and implant placement in maxillary sinus lift procedures, suggesting that 4 months of healing with sintered xenogeneic bone substitute is sufficient for effective osseointegration. Additional clinical trials are necessary to evaluate different bone substitutes and optimize healing time for implant-supported rehabilitation protocols.

The histomorphometric findings demonstrated a significant increase in neoformation and bone tissue maturation over time, indicating a favorable biological response to the use of sintered bovine bone substitute. Comparing the healing periods of 4 and 8 months, it was observed that a longer interval resulted in greater bone density and collagen fiber organization, highlighting the importance of time in determining the quality of bone regeneration in maxillary sinus lift procedures.

## Data Availability

The research data are available within the article
